# Immune and stromal cell interaction in Sjögren's disease

**DOI:** 10.1016/j.ebiom.2026.106205

**Published:** 2026-03-19

**Authors:** R. Hal Scofield

**Affiliations:** Arthritis & Clinical Immunology Program, Oklahoma Medical Research Foundation, Department of Medicine, College of Medicine, University of Oklahoma Health Sciences Center, Oklahoma City US Department of Veterans Affairs Medical Center, 825 NE 13th Street, Oklahoma City, OK, 73104, USA

Sjögren's disease is an autoimmune illness that affects the salivary and lacrimal glands. In addition, the condition is also systemic with autoantibodies binding proteins expressed in virtually all cell types. Further, at least half of patients have disease beyond these exocrine glands, including arthritis, pulmonary fibrosis, renal tubular acidosis, vasculitis, and neurological involvement, among others.^1^ The key features of the illness that indicate autoimmunity are autoantibodies to Ro (or SSA) antigens (Ro60 and Ro52, AKA TROVE2 and TRIM21) and La (or SSB) as well as lymphocytic infiltrate of the salivary and lacrimal glands.

This infiltrate consists of focal collections of T and B lymphocytes and frequently contains tertiary lymphoid structures such as germinal center organization with follicular T cells.^2^, ^3^, ^4^ B lymphocytes in the salivary gland produce autoantibodies.^5^^,^^6^ One can certainly read that the lymphocytic infiltration found in Sjögren's leads to the destruction of the gland and subsequent dysfunction and sicca symptoms. However, in fact, on routine histological examination, in many patients the majority of the salivary gland is normal in appearance. Thus, one of enigmas of the disease is why glands that are not destroyed, do not function.

The answer to this question may in part lie in the effect of the lymphocytic infiltration on glandular stromal cells. The latter increasingly have been found important for the pathogenesis of Sjögren's disease.^7^ There are clear abnormalities of the salivary epithelial cells, including expression of HLA class II molecules and production of inflammatory cytokines.^8^ Thus, a critical research question is the interaction of the immune infiltrate with the resident stromal cells.

In the recent issue of *eBioMedicine*, Li and colleagues have approached this question through spatial transcriptomics.^9^ This work demonstrated there are spatially organized networks of immune and epithelial cells in the salivary glands of Sjögren's disease patients—a landscape or architecture of immune-stromal cell interaction, if you will. In particular, previously unappreciated stromal cells were found within lymphocytic infiltrates; namely, a CCL2^high^ fibroblast and ACKR1^high^ endothelial cell. These two cells were correlated with each other and spatially co-localized within and around lymphocyte foci. In addition, while only a handful of Sjögren's patients were studied, these cells were quantitatively associated with disease activity and focus score. Crosstalk between these cells and immune cells within foci was identified by examination of expression of cytokines and chemokines as well as their respective receptors. For instance, CXCL12 was expressed by endothelial cells and fibroblasts while its receptor CXCR4 was expressed on T and B cells.

This work also implicated particular metabolic pathways in salivary gland stromal cells. Within lymphocytic foci these investigators identified altered lipid metabolism (especially phosphatidylinositol signaling), which was spatially associated with lymphocyte activation as well as interferon production. There were also multiple metabolic abnormalities present in epithelial cells within the lymphocytic foci. Perhaps the most interesting of these was suppressed glycerolipid metabolism, which was associated with poorer glandular function.

There are no effective disease-modifying therapies for Sjögren's disease, although repurposing the combination of hydroxychloroquine and leflunomide has promising data.^10^ However, there are five agents that have either completed US FDA Phase III trials or have such trials ongoing. Some of these agents (or even 1 of them) coming to the market with an indication for Sjögren's will mark a new era for the disease. Nonetheless, there is still much work to be done to understand the pathogenesis. Unraveling the immune and stromal cell interactions that may underlie enigmatic glandular dysfunction will be another important step in improving understanding in a way that eventually affects patients with Sjögren's disease.

## Contributors

RHS was the sole author of this work. As author, he read and approved the final version.

## Declaration of interests

The author has received honoraria payments for participation in an advisory board by Jensen Pharmaceuticals and Veloxis Pharmaceuticals as well as from IVQIA for membership in a clinical trial data safety monitoring board. The author is local site PI for a clinical trial of nipocalamab in Sjögren's disease with a contract from Jensen Pharmaceuticals to the OMRF.
